# Beyond the hatchery: testing managed wetland impoundments as semi-natural rearing habitat for endangered Delta Smelt

**DOI:** 10.1093/conphys/coag076

**Published:** 2026-10-01

**Authors:** Florian Mauduit, Dennis E Cocherell, Shawn Acuña, Nann A Fangue

**Affiliations:** Fish Conservation Physiology Laboratory, Department of Wildlife, Fish and Conservation Biology, University of California, Davis, 1 Shields Avenue Davis CA95616, USA; Fish Conservation Physiology Laboratory, Department of Wildlife, Fish and Conservation Biology, University of California, Davis, 1 Shields Avenue Davis CA95616, USA; Metropolitan Water District of Southern California, 700 North Alameda Street Los Angeles, CA 90012-2944, USA; Fish Conservation Physiology Laboratory, Department of Wildlife, Fish and Conservation Biology, University of California, Davis, 1 Shields Avenue Davis CA95616, USA

**Keywords:** Conservation aquaculture, Delta Smelt, floating wetlands, hypoxia, managed wetlands, temperature, zooplankton

## Abstract

Conservation of the endangered Delta Smelt (*Hypomesus transpacificus*), endemic to the San Francisco Estuary (CA, USA), increasingly relies on captive propagation. Yet fish reared under standard hatchery conditions may not develop the behaviours and physiological traits necessary for survival following release. Semi-natural rearing environments that expose fish to natural environmental variability while maintaining protection from predators and other risks can address this challenge. We evaluated the potential of managed wetland impoundments equipped with floating wetland as semi-natural rearing habitats for Delta Smelt before supplementation. Three field deployments were conducted on Bouldin Island between winter 2022 and spring 2023. We monitored survival, growth, glycogen reserves, cortisol concentrations, environmental conditions and zooplankton communities. Survival was high during winter and early spring but declined later in spring as temperatures approached ca. 20–23°C and repeated low-oxygen events occurred. Growth responses varied among deployments and treatments. Fish exhibited depletion of glycogen reserves during field exposure, consistent with increased energetic demand or adjustment to natural foraging conditions. Cortisol concentrations increased over time, indicating rising physiological challenge as environmental conditions became more demanding. Floating wetlands slightly reduced diurnal fluctuations in temperature and dissolved oxygen but did not improve fish performance. Zooplankton communities were dominated by suboptimal prey taxa, suggesting prey quality may have constrained energetic condition. These results suggest that managed impoundments can function as transitional habitats that help prepare hatchery-reared Delta Smelt for natural conditions before release. However, their effectiveness will depend on seasonal timing, prey availability and water-quality conditions.

## Introduction

The Delta Smelt (*Hypomesus transpacificus*), a pelagic osmerid fish endemic to California’s upper San Francisco Bay-Delta estuary (Moyle, 2002; Bennett, 2005), is highly susceptible to environmental variability due to its 1-year life cycle and low fecundity (Hobbs *et al.*, 2017). Once abundant (Bennett, 2005), its population has sharply declined since the 1980s (Baxter *et al.*, 2015) and it is listed as an endangered species under the California Endangered Species Act and as threatened under the US Endangered Species Act, with recommendations for reclassification as endangered (White, 2020). Multiple stressors such as habitat loss and alteration, reduced freshwater flow due to water exports, interactions with invasive species such as predation and competition, and significant changes in the food web have driven this decline (Moyle, 2002; Hobbs *et al.*, 2017). Delta Smelt occupy a mid-trophic position in the estuarine food web, feeding on small zooplankton while serving as prey for piscivorous fish and birds. Their collapse points to the need for conservation that sustains the estuary’s function (Bennett, 2005).

In response to the species’ decline and risk of extinction, a captive propagation program was initiated in 1996, and a genetically managed refuge population has been maintained at the University of California Davis Fish Conservation and Culture Laboratory (FCCL) since 2008 (Lindberg *et al.*, 2013; Finger *et al.*, 2018). The program aims to prevent the species’ extinction, support research and supplement the wild population with hatchery-raised fish (Lindberg *et al.*, 2013; Hobbs *et al.*, 2017). To preserve genetic diversity and reduce domestication effects, wild fish are added to the broodstock each year, and pairings are selected to maximize outcrossing (Finger *et al.*, 2018). Wild fish have become increasingly scarce, making reliance on wild broodstock tenuous. At the same time, potential adaptation to captivity over successive generations remains of concern. For example, crosses with one wild parent showed lower reproductive success than crosses of two hatchery parents (Finger *et al.*, 2018), although the mechanisms underlying this pattern remain uncertain and may include differences in parental condition, gamete quality or responses to captive breeding. A recent critical thermal tolerance (CTmax) assessment reports higher CTmax in low-domestication fish and lower CTmax in high-domestication lines (Griffiths *et al.*, 2026), with implications for selection and stress tolerance of the fish produced.

Hatchery environments can inadvertently select for traits that may be advantageous in captivity but maladaptive in the wild, leading to reduced fitness upon release. For instance, studies have shown that hatchery-reared salmonids often exhibit lower reproductive success in natural environments compared to their wild counterparts, sometimes within just one or two generations of captive breeding (Araki *et al.*, 2008). Additionally, research on steelhead trout (*Oncorhynchus mykiss*) indicates that offspring of first-generation hatchery fish grow faster in hatchery settings but have lower survival rates in natural stream environments, suggesting rapid domestication selection (Blouin *et al.*, 2021). In particular, fish raised in an environment free of predators and with plentiful feed often exhibit reduced anti-predator behaviours and weaker foraging skills (Brown and Day, 2002). Although careful hatchery practices can mitigate certain domestication pressures, they cannot eliminate them entirely (Frankham, 2008).

This general challenge has also surfaced in numerous cultured–wild species interactions (Carson *et al.*, 2016; Osborne *et al.*, 2020), revealing how genetic and behavioural shifts can accrue once hatchery fish mix with their wild counterparts. Recognizing the potential risks of inadvertently domesticating species intended for population supplementation, conservation biologists stress the importance of bridging the gap between captive and wild environments to preserve the ‘wildness’ of cultured fish (Brown and Day, 2002). Complementing this perspective, indigenous knowledge systems have long valued wildness as the maintenance of behavioural and ecological integrity. Recent co-produced work in conservation physiology with indigenous partners highlights approaches such as streamside and in-stream salmon rearing that preserve natural cues and behaviours while meeting management needs (Cooke *et al.*, 2025). By implementing rearing practices that simulate natural conditions, such as semi-natural ponds, structural habitat complexity and controlled predator exposure, hatchery programs could reduce domestication pressures and better prepare fish for release into their native habitats. However, only a few programs have fully addressed the ecological and evolutionary challenges of conservation hatcheries by employing more natural settings (Marsh *et al.*, 2015). Notable examples include programs for the endangered Razorback Sucker (*Xyrauchen texanus*) and Rio Grande Silvery Minnow (*Hybognathus amarus*), in which wild spawning is permitted in natural habitats, followed by the collection of wild-fertilized eggs or larvae for hatchery rearing (Osborne *et al.*, 2012; Carson *et al.*, 2016). Such semi-natural methods have proven effective: long-term data indicate that captive-reared Razorback Suckers and Silvery Minnows achieve growth and survival rates comparable to their wild conspecifics (Osborne *et al.*, 2020). Critically, these programs have managed to conserve genetic diversity despite population bottlenecks in the wild (Dowling *et al.*, 2014; Carson *et al.*, 2016). Although the present study was not conducted as a formal co-produced project with indigenous partners, semi-natural rearing approaches are compatible with conservation perspectives that emphasize maintaining behavioural and ecological integrity. Future refinement of Delta Smelt conservation rearing could benefit from co-developed management frameworks that integrate ecological, physiological and indigenous knowledge perspectives (Cooke *et al.*, 2025).

Recent pilot studies demonstrated that Delta Smelt can be reared *in situ* under environmental conditions akin to the wild. For example, field trials housing Delta Smelt in floating enclosures on the Sacramento River observed survival rates exceeding 94% over 4 weeks (Baerwald *et al.*, 2023). During this time, Delta Smelt rapidly transitioned from hatchery feed to wild zooplankton, demonstrating a rapid return to natural foraging. Growth results varied across sites, highlighting the importance of site-specific factors such as water quality and prey abundance. Together, these trials point to promising rearing sites within the Delta Smelt range, including managed floodplain wetland impoundments where floating wetland modules can be deployed.

Rearing fish in managed floodplain wetland impoundments is a common practice in commercial aquaculture and conservation contexts, as these habitats can enhance fish growth and survival by providing warmer temperatures, reduced water velocities and abundant natural prey resources (Corline *et al.*, 2017; Katz *et al.*, 2017). Such approaches have been particularly successful for species like juvenile Chinook Salmon (*Oncorhynchus tshawytscha*), which show increased growth rates when reared in ephemeral floodplain habitats (Jeffres *et al.*, 2008). Similar semi-natural strategies have proven effective in conservation programs for the endangered Razorback Sucker (*X. texanus*) and Rio Grande Silvery Minnow (*H. amarus*), demonstrating long-term success in preserving genetic diversity and facilitating high post-release survival (Dowling *et al.*, 2014; Marsh *et al.*, 2015; Carson *et al.*, 2016; Osborne *et al.*, 2020; Yackulic *et al.*, 2022; Archdeacon *et al.*, 2023). In tandem with these conservation aquaculture initiatives, habitat restoration and engineering solutions are under development to bolster Delta Smelt in their natural setting. One emerging technique is the use of floating wetlands, buoyant artificial islands supporting wetland vegetation. In a recent mesocosm study, floating peat platforms in the Delta were shown to remain buoyant and accumulate biomass at rates mirroring those in non-tidal managed wetlands (Deverel *et al.*, 2024). Notably, in treatments with ~45% open water, the peat islands expanded laterally by 21% per year and demonstrated vertical accretion of 5.5–8.6 cm/year. Accordingly, managed impoundments and floating wetland technology both present promising means of rearing Delta Smelt in near-natural conditions, enabling continued monitoring and safeguarding. Critically, floating wetlands develop into self-sustaining marsh-like habitats, boosting zooplankton production (Corline *et al.*, 2017) and buffering fluctuations in temperature and dissolved oxygen (DO) via shading and oxygenation processes (Hatton *et al.*, 1983; de Moraes *et al.*, 2023). Consequently, their introduction in Delta channels or impoundments may enhance water quality and foraging opportunities for pelagic fish such as the Delta Smelt (Deverel *et al.*, 2024). However, managed impoundments may also present challenges for Delta Smelt rearing, including seasonal warming, episodic low DO and potential mismatches between available zooplankton communities and preferred Delta Smelt prey. Used with habitat enhancements like floating wetlands, managed impoundments can better prepare Delta Smelt for release by improving survival, supporting natural foraging and advancing supplementation objectives.

We conducted three consecutive *in situ* deployments on Bouldin Island, CA, to test how seasonal conditions and habitat features influence Delta Smelt rearing in managed impoundments. Deployments 1 (winter) and 2 (spring) evaluated sub-adult survival and performance across two impoundments, whereas Deployment 3 compared enclosures placed adjacent to floating-wetland modules with enclosures in nearby open water. In each deployment, Delta Smelt were held in enclosed cages that exposed fish to ambient zooplankton and environmental variability while maintaining protection within the enclosures. Water temperature and DO were monitored, and fish survival and growth were measured to evaluate rearing performance. Glycogen reserves were measured as indicators of stored energy availability (French *et al.*, 1983), while whole-body cortisol was measured as an indicator of physiological stress and energy mobilization (Barton and Iwama, 1991; Mommsen *et al.*, 1999; Barton, 2002).

## Materials and methods

### Study design and location

The study involved three distinct deployments aimed at assessing the suitability of managed wetland impoundments and habitat enhancements for rearing Delta Smelt. Deployments 1 and 2 were conducted within two adjacent managed wetland impoundments (designated ‘West’ and ‘East’) located on Bouldin Island in the Sacramento–San Joaquin Delta, CA (Fig. 1A). In this manuscript, we use ‘impoundment’ to refer to shallow, leveed wetland cells on Bouldin Island that are hydrologically managed for habitat and restoration outcomes (e.g. vegetation establishment, improved water quality). During the study, approximate water depths were 2.7–3.0 m in the West impoundment and 3.0–3.7 m in the East impoundment. These cells are hydrologically distinct from adjacent tidal channels and function as managed wetlands rather than production aquaculture ponds or reservoirs. The East and West impoundments used here are part of ongoing wetland management and restoration activities on Bouldin Island. Specifically, Deployment 1 evaluated Delta Smelt survival and performance under colder winter conditions from November 2022 to 4 January 2023, whereas Deployment 2 assessed these parameters during warmer spring conditions from 6 March to 3 May 2023. Deployment 3, conducted exclusively in the West impoundment from 13 March to 13 April 2024, specifically examined the benefits of incorporating floating wetland modules. Within the West impoundment during Deployment 3, two experimental treatments were established: one set of fish enclosures was placed adjacent to floating wetland modules, and a control set of enclosures was located ~50 m away in open-water conditions (Fig. 1B). The floating wetland modules consisted of buoyant vegetated peat mats designed to mimic natural marsh habitat structure, as described by Deverel *et al.* (2024).

**Figure 1:**
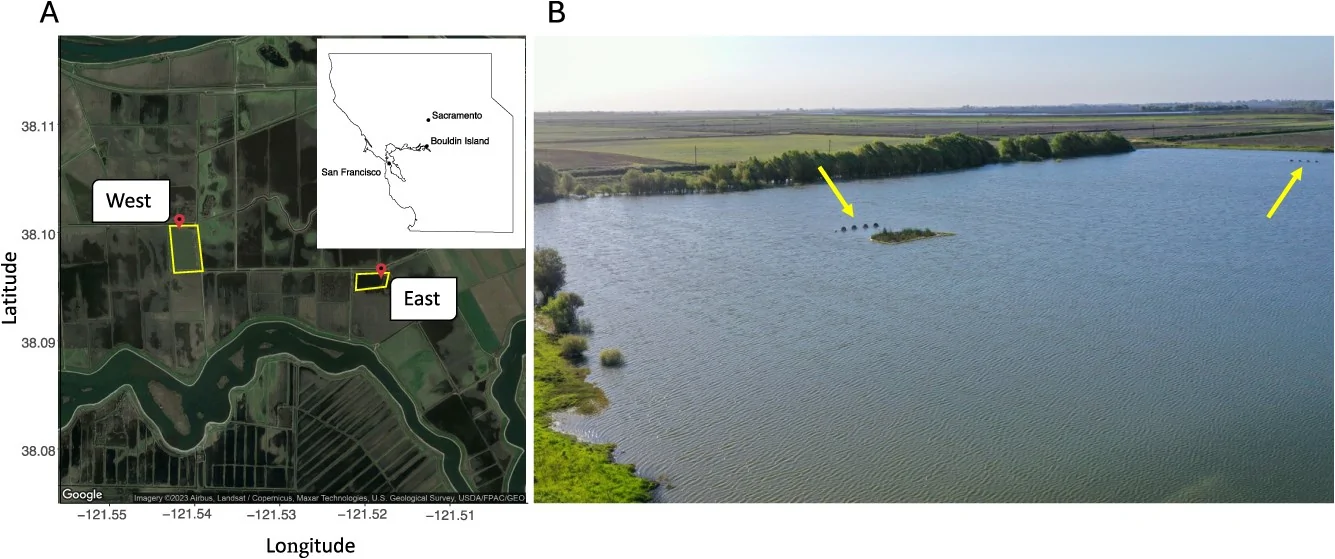
(A) Map of the study area on Bouldin Island, located within the Sacramento–San Joaquin Delta, CA. The image highlights the locations of the two managed wetland impoundments (West and East) utilized during experimental deployments. Deployments 1 and 2 occurred within these impoundments to evaluate Delta Smelt survival and performance under winter and spring conditions, respectively. Deployment 3 was specifically conducted in the West impoundment to investigate the effects of floating wetland modules on Delta Smelt rearing outcomes. The coordinates are presented in decimal degrees. The inset map shows the broader geographical context of Bouldin Island relative to major regional landmarks. (B) Picture of the West impoundments during the third deployment showing the floating island. Enclosures positioned near and far from the floating islands are indicated by the arrows. © Russell Ryan, Reclamation District 756 Trustee.

### Enclosures design and setup

Delta Smelt enclosures were custom constructed following enclosure designs previously developed and tested for Delta Smelt field deployments (Baerwald *et al.*, 2023; Davis *et al.*, 2024; Gille *et al.*, 2026). Each enclosure was cylindrical, measuring 1.0 m in diameter and 1.3 m in height, and built from perforated steel mesh with ~40% openness (mesh hole diameter: 3.18 mm). This enclosure design was intended to allow moderate water exchange and natural zooplankton prey availability while preventing fish escape, minimizing impingement risk during higher flows, and excluding predators. Although zooplankton were not sampled directly within individual enclosures in the present study, previous Delta Smelt enclosure studies using similar designs have shown that fish can access and consume wild zooplankton prey in the enclosures (Baerwald *et al.*, 2023; Davis *et al.*, 2024; Gille *et al.*, 2026). Each enclosure featured a secure lid to prevent avian predation, and all were routinely checked to ensure structural integrity and continued functionality. Enclosures were deployed in clusters of three, anchored securely in place and tethered in a linear configuration using 3-m lengths of polyvinyl chloride (PVC)-sleeved steel cable to maintain consistent spacing between cages.

For Deployments 1 and 2, one cluster consisting of three enclosures was placed in the West impoundment, and an additional cluster of three enclosures was placed in the East impoundment. During Deployment 3, two clusters (each comprising three enclosures) were positioned exclusively within the West impoundment, one cluster adjacent to (‘Near’) and another 50 m away from (‘Far’) the floating wetland modules. Because enclosures within each treatment experienced the same ambient impoundment conditions, individual cages were treated as the unit of replication in statistical analyses.

### Environmental conditions monitoring

Throughout the study, water temperature and DO were continuously monitored within the experimental enclosures. Specifically, for all three deployments, DO and temperature loggers (HOBO U26-001, Onset) were placed within one representative enclosure per treatment group (i.e. West and East impoundments for Deployments 1 and 2; enclosures Near and Far from the floating wetland modules for Deployment 3). Additionally, temperature loggers (HOBO UA-001-64) were placed in every enclosure to verify that temperature conditions remained consistent among enclosures within the same treatment group. DO and temperature measurements were recorded every 10 min. Enclosures were also visually inspected weekly to confirm structural integrity, equipment functioning and overall fish condition.

### Fish acquisition, transport and sampling

Delta Smelt used in this study were sourced from the University of California Davis FCCL captive refuge population. Fish used across the three deployments came from the 14th, 15th and 16th captive generations of the conservation breeding program, depending on deployment cohort. For each deployment, a total of 360 Delta Smelt juveniles/sub-adults were transported from the FCCL to Bouldin Island in insulated 120-l containers, each containing 60 fish in water supersaturated with oxygen (130% of air saturation) at 5 ppt salinity, according to established FCCL transport protocols. Fish size differed among deployments because deployments occurred at different times of year and used different cohorts. At the start of Deployment 1, fish averaged 1.03 ± 0.22 g and 47.8 ± 0.7 mm fork length. At the start of Deployment 2, fish averaged 1.93 ± 0.15 g and 60.1 ± 0.7 mm fork length. At the start of Deployment 3, fish averaged 3.27 ± 0.22 g and 73.2 ± 1.5 mm fork length. These size differences indicate that fish used in Deployments 2 and 3 were larger and likely more developmentally advanced than those used in Deployment 1. These differences in size and seasonal timing were important for interpreting deployment-specific responses, particularly the spawning activity and post-spawning mortality observed during Deployment 2. Transport duration was ~1 h. Upon arrival at the impoundments, transport water was partially mixed with ambient impoundment water over ~1 min to reduce abrupt differences in temperature and water chemistry before fish were gently released into their respective enclosures. No mortality was observed during the transportation or stocking processes. Each enclosure was stocked with 60 fish, a density previously shown to minimize crowding stress in Delta Smelt (Baerwald *et al.*, 2023).

Sampling occurred at specific intervals to assess survival, growth and physiological condition. Survival was assessed for all deployments by counting live fish remaining in each enclosure, and growth was assessed from fork length and wet mass measurements of sampled fish. For Deployment 1, fish were measured at the hatchery prior to deployment to characterize initial size (*n* = 20) and from enclosures at the end of the deployment to assess growth (*n* = 20 fish per enclosure; 3 enclosures × 2 impoundments = 120 fish). For Deployment 2, fish were weighed, measured and terminally sampled at the hatchery prior to deployment and from the East and West impoundments at 6 and 13 weeks post-deployment to assess growth and physiological condition; whole-body glycogen was analysed using 9 fish per group and time point, and whole-body cortisol was analysed using 10 fish per group and time point. For Deployment 3, fish were weighed, measured and terminally sampled at the hatchery prior to deployment and from Near and Far enclosures at the conclusion of the 4-week deployment to assess growth and liver glycogen; liver glycogen was analysed using 24 fish per sampling group (T0, Near, and Far).

At each enclosure sampling event, fish were collected using a dip net without actively chasing individual fish. During Deployment 2, the first scoop from each enclosure was prioritized for cortisol analysis to minimize capture- or handling-induced effects on cortisol measurements. Fish collected for physiological analyses were immediately euthanized using an overdose of buffered tricaine methanesulfonate (500 mg/l MS-222), measured for fork length (nearest 1 mm) and wet mass (0.01 g), and flash-frozen as quickly as possible to preserve tissue integrity. When additional fish were needed to complete the planned sample size, a second scoop was collected and used for glycogen analysis, which was considered less sensitive to short-term capture stress than cortisol. Fish were processed individually following euthanasia. Mortalities observed during routine enclosure checks were removed and recorded when present.

### Physiological assessments

#### Glycogen and cortisol sample preparation

Whole-body samples collected during the second deployment were homogenized to extract glycogen and cortisol. Prior to homogenization, fish heads were removed to avoid potential interference in cortisol and metabolite measurements resulting from brain tissues, in accordance with established methods (Hasenbein *et al.*, 2013; Pasparakis *et al.*, 2022). The headless fish bodies were ground into a fine powder using a mortar and pestle over liquid nitrogen, after which the homogenate was divided into two aliquots for independent glycogen and cortisol extraction. All powdered subsamples were weighed consistently within a range of 100–130 mg.

In the third deployment, liver tissue was sampled and flash frozen in liquid nitrogen to quantify hepatic glycogen levels. Liver glycogen was assessed as a targeted indicator of short-term energy storage and nutritional status (French *et al.*, 1983; Dhayalan *et al.*, 2024).

#### Glycogen extraction and assay

Whole-body powder samples collected from fish during the second deployment were extracted by sonicating the powder on ice in 500 μl of 8% perchloric acid, using four 6-s bursts with a Micro Ultrasonic Cell Disrupter (Model 120 Sonic Dismembrator; Fisher Scientific) set on its highest setting. After sonication, homogenates were centrifuged at 10000 × *g* for 10 min at 4°C. The resulting supernatant was neutralized by adding 40 μl of 3 M potassium carbonate (K₂CO₃). Neutralized extracts underwent an additional centrifugation step (10 000 × *g*, 10 min, 4°C) to remove any remaining precipitates, after which they were stored at −80°C until analysis.

A similar extraction procedure was applied to liver tissues collected during the third deployment. Each liver was individually weighed and transferred to a 2-ml tube before immediate sonication on ice in 8% perchloric acid to reach a homogenate concentration of 1 mg tissue/20 μl, again using four 6-s sonication bursts on the highest setting (Micro Ultrasonic Cell Disruptor). After sonication, the homogenate was centrifuged at 10000 × *g* for 10 min at 4°C, and the resulting supernatant was neutralized with 40 μl of 3 M K₂CO₃. The neutralized extracts underwent a second centrifugation to remove additional precipitates, and the final clear supernatants were stored at −80°C until analysis.

Glycogen concentrations were determined using an enzymatic assay kit (MAK016, Sigma). Neutralized extracts were assayed according to the manufacturer’s instructions, with absorbance measured at the appropriate wavelength (e.g. 570 nm) on a microplate reader (Infinite M200, Tecan Life Sciences). Free glucose was measured in parallel by omitting the hydrolysis enzyme mix from the reaction, and these values were subtracted from total glucose values obtained after hydrolysis to calculate glycogen-specific concentrations. All samples were run in duplicate to ensure analytical precision. Coefficients of variation between duplicates were all below 10%.

#### Cortisol extraction and assay

Cortisol extraction protocols followed methods previously applied to whole-body cortisol analysis in Delta Smelt and related smelt studies (Hasenbein *et al.*, 2013; Pasparakis *et al.*, 2022). Powdered fish tissues sampled during the second deployment were homogenized with 1 ml of 1× phosphate-buffered saline (PBS; P5368, Sigma-Aldrich, St. Louis, MO, USA) using a polytron-type homogenizer (PowerGen 125, Fisher Scientific) over ice for 15 s. The homogenates were subsequently mixed with 2.5 ml of diethyl ether (346136-1 L, Sigma-Aldrich), vortexed for 1 min, and centrifuged at 3800 rpm for 10 min at 4°C. This ether extraction process was repeated twice more (three washes total), with supernatants from each wash combined into a separate tube and evaporated completely in a fume hood. Dried samples were resuspended in either 100 μl or 175 μl of 1× PBS, transferred into 1.5-ml tubes, and stored at −80°C.

Cortisol concentrations were determined using an enzyme-linked immunosorbent assay kit (Salimetrics, Carlsbad, CA, USA), according to the manufacturer’s guidelines. Samples were randomized during extraction and assay processing to minimize potential batch or extraction-order effects. Samples were analysed in duplicate, and cortisol concentrations (ng/dl) were calculated using a four-parameter logistic regression model. Concentrations were then normalized to sample mass to yield final cortisol concentrations in ng/g (nanogram cortisol per gram of fish tissue). A spike recovery test was not conducted to quantify cortisol extraction efficiency; therefore, cortisol values were interpreted as relative comparisons among treatment groups and time points. Coefficients of variation between duplicates were all below 10%.

### Zooplankton monitoring

Zooplankton data were downloaded directly from USGS ScienceBase as CSV files (catalogue items 63c870c3d34e06fef14f3537 and 672d12f8d34ea9e45088dfe9). Data from ponds on islands of the Sacramento–San Joaquin Delta (November 2021–November 2022) were downloaded from Buxton *et al.* (2023), and data from the Floating Wetland Experiment in the West Pond on Bouldin Island (30 January to 30 April 2024) were downloaded from Buxton *et al.* (2025). Both datasets included monthly zooplankton samples collected with a Schindler-Patalas 12 L plankton trap fitted with a 200-ml Dolphin bucket and 61-μm mesh. Samples were preserved in 10% formalin and processed by EcoAnalysts, Inc., following their standard operating procedures for taxonomic identification, enumeration and biomass calculations. To ensure relevance to our fish deployments, we only used USGS samples that were co-located and time aligned with our study sites. The 2021–22 island-pond data characterize typical zooplankton communities in Delta island ponds used for managed wetlands, while the 2024 dataset was collected in the West Pond on Bouldin Island during the same period as Deployment 3, providing a site- and period-specific index of prey availability for our enclosures. These impoundments are not part of a routine long-term USGS monitoring network; rather, they were sampled specifically for the published USGS pond and floating-wetland projects cited above. Accordingly, the USGS series serve as an appropriate, independent measure of zooplankton prey available to Delta Smelt within the exact waterbodies and dates of interest.

### Statistical analysis

All analyses were conducted in GraphPad Prism (v10.4.2), figures were prepared in R (4.3.0) using ggplot2, and two-tailed *α* was set to 0.05. Survival in Deployments 1 and 3 was compared between treatments using Welch’s two-sample *t*-tests. When assumptions of normality or homogeneity of variance were not met, nonparametric tests were used; for Deployment 2, survival across the 6- and 13-week time points was analysed using a mixed-effects model with time and impoundment as fixed effects and enclosure included as a random effect. Growth was assessed with Welch’s *t*-tests in Deployments 1 and 3 and with a two-way analysis of variance (ANOVA) (time × impoundment) in Deployment 2, followed by adjusted *post hoc* contrasts as appropriate. Metabolites were evaluated as follows: whole-body cortisol in Deployment 2 was tested with a mixed-effects model (significant main effect of time), while glycogen and other non-normal datasets were analysed with rank-based tests and multiplicity-corrected pairwise comparisons when indicated; liver glycogen in Deployment 3 was compared among groups using nonparametric methods due to distributional assumptions. Zooplankton biomass was summarized descriptively as relative contributions of dominant taxa, and we did not perform formal multivariate community analyses due to limited replication and strong dominance by a few taxa.

## Results

### Environmental conditions

In the initial winter deployment, temperatures in the East impoundment ranged from 8.20°C (08:30 on 20 December 2022) to 15.30°C (15:00 on 21 November 2022), whereas the West impoundment recorded temperatures between 8.00°C (07:00 on 20 December 2022) and 12.40°C (14:00 on 11 November 2022, Fig. 2A). Mean diurnal temperature fluctuations, calculated as daily maximum minus daily minimum, averaged 0.59 ± 0.08°C in the East impoundment and 0.70 ± 0.05°C in the West impoundment. The East impoundment exhibited greater maximum daily fluctuations (up to 3.00°C) compared to the West (up to 1.60°C).

**Figure 2:**
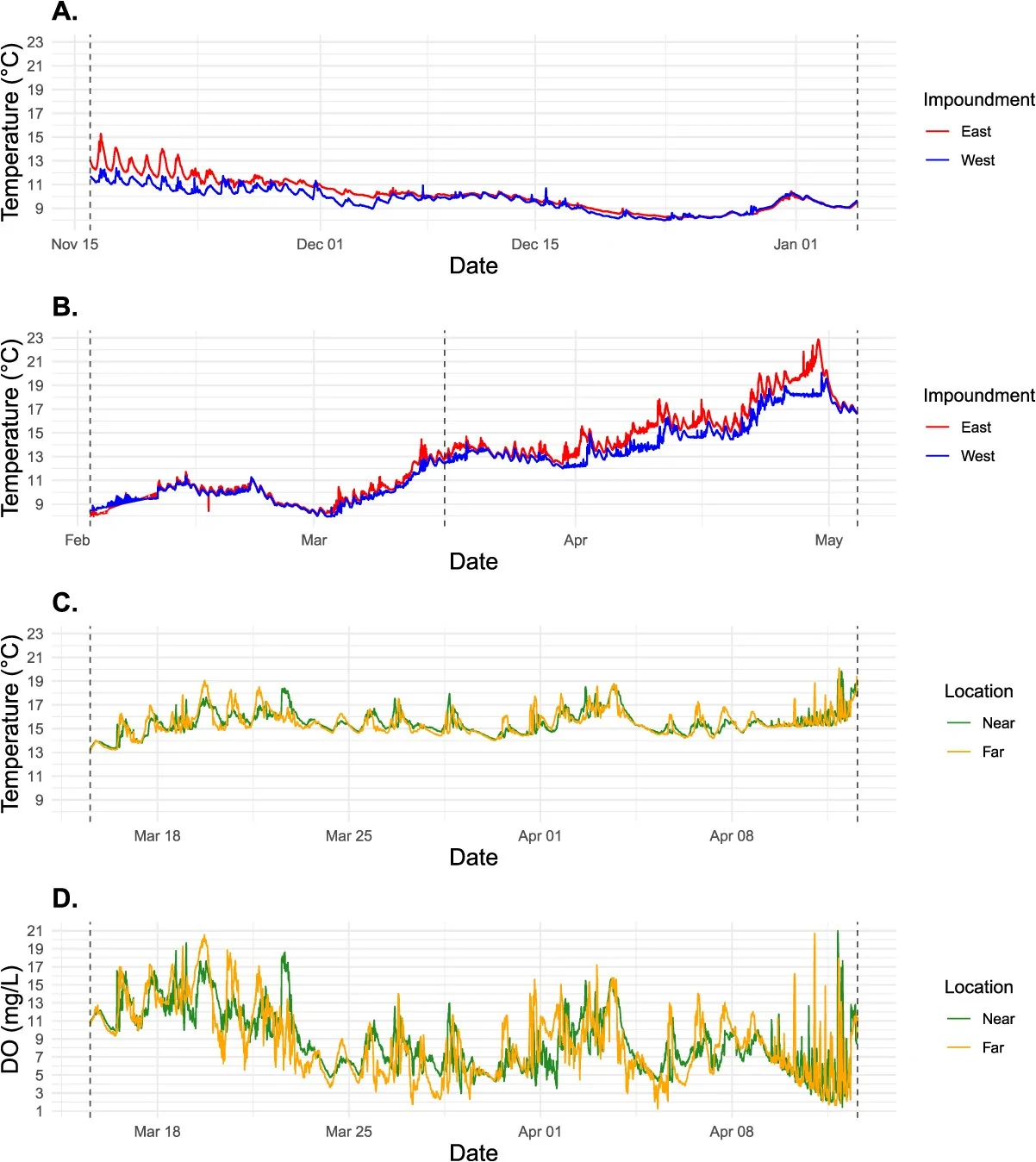
Temporal dynamics of water temperature and DO during three successive deployments. The top 3 panels display water temperature time series from the first, second and third deployments, respectively. Panels A and B show temperature (°C) data from the East (red) and West (blue) impoundments during the first and second deployments, respectively. Panel C presents temperature data from the third deployment, measured at two locations within the West impoundment: ‘Near’ the floating wetland (green) and ‘Far’ from it (orange). The bottom panel (D) depicts DO (mg/l) levels recorded during the third deployment. Dashed lines indicate the sampling points.

Throughout the second spring deployment, temperature ranges broadened considerably. Temperatures in the East impoundment ranged from 7.90°C (06:30 on 6 March 2023) to 22.80°C (15:30 on 2 May 2023), while the West impoundment recorded temperatures between 7.90°C (07:00 on 5 March 2023) and 20.10°C (15:00 on 2 May 2023, Fig. 2B). Mean diurnal fluctuations increased, averaging 1.11 ± 0.07°C in the East impoundment and 0.81 ± 0.05°C in the West impoundment, with similar maximum fluctuations observed in both impoundments (2.70°C in the East and 2.80°C in the West).

For the third deployment, continuous monitoring of water temperature and DO was conducted at two sites within the West impoundment: one near the floating islands and one at a control site (far from the islands). Temperature gradually increased over the monitoring period. At the site near the floating islands, temperatures ranged from a minimum of 13.26°C (13:00 on 15 March 2024) to a maximum of 19.84°C (00:10 on 12 April 2024). At the control site, temperatures varied from 13.10°C (13:00 on 15 March 2024) to a maximum of 20.08°C (22:10 on 11 April 2024, Fig. 2C). Mean diurnal temperature variations were 1.99 ± 0.17°C near the floating islands and 2.50 ± 0.19°C at the control site. DO concentrations exhibited substantial variability at both sites. Near the floating islands, DO ranged from a maximum of 20.98 mg/l (21:00 on 11 April 2024) to a minimum of 1.44 mg/l (00:20 on 12 April 2024, Fig. 2D). At a 6 mg/l threshold, DO remained below this level for ~148.5 h across 71 events, with the longest continuous hypoxic episode lasting ~22.17 h (from 13:30 on 29 March to 11:20 on 30 March 2024). At a more severe threshold of 3 mg/l, cumulative hypoxia duration near the islands totaled 8.50 h across 11 events, with the longest continuous episode lasting ~2.5 h (from 14:10 to 16:30 on 11 April 2024.). At the control site, DO ranged from a maximum of 20.69 mg/l (00:40 on 11 April 2024) to a minimum of 1.25 mg/l (01:20 on 5 April 2024). Using the threshold of 3 mg/l, total hypoxia duration was 22.67 h across 22 events, with the longest continuous hypoxic period lasting ~3.67 h (from 05:40 to 09:10 on 28 March 2024).

### Survival

During the first deployment, conducted over a 6-week period in winter, survival rates were 100 ± 0% in the East impoundment and 96 ± 3% in the West impoundment (Fig. 3A). A *t*-test confirmed that this difference was not statistically significant (*P* = 0.261).

**Figure 3:**
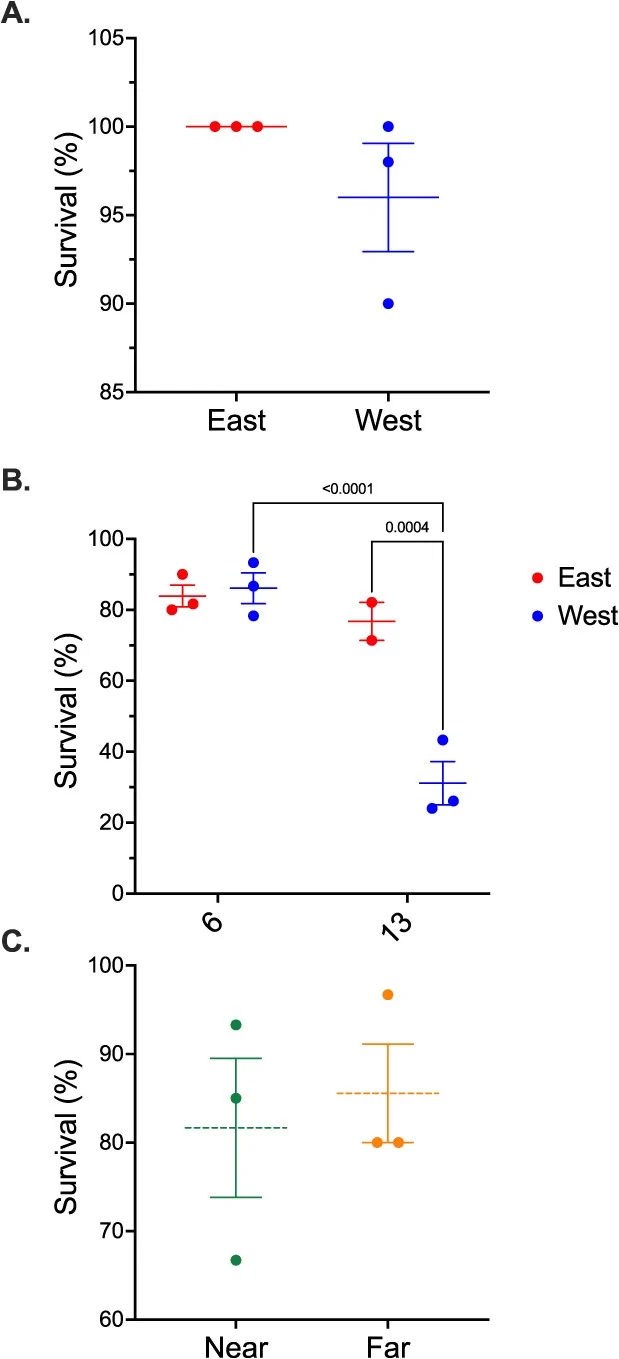
Survival of Delta Smelt across three deployments. Panels A–C show survival (%) in each experimental condition. Panel A presents survival from the first deployment, comparing enclosures located in the East and West impoundments. Panel B shows survival from the second deployment, measured at 6 and 13 weeks post-deployment in the East and West impoundments. Panel C displays survival from the third deployment, comparing enclosures placed near the floating wetland modules (‘Near’) and ~50 m from the wetlands (‘Far’), both located within the West impoundment. Points represent enclosure-level survival values, with three replicate enclosures per treatment/time point. Horizontal lines and error bars represent the mean ± SEM calculated across enclosure-level values. Statistical comparisons between groups are annotated with corresponding *P*-values.

The second deployment, conducted in spring over a 13-week period, included survival assessments at two time points. At the 6-week mark, fish in the East impoundment showed a survival rate of 83.9 ± 3.0%, while those in the West impoundment had a survival rate of 86.1 ± 4.3%. No significant difference was detected between the two sites at this point (mixed model, *P* = 0.935; Fig. 3B). By the end of the deployment, survival in the East impoundment declined to 76.8 ± 5.4%, while survival in the West impoundment dropped more substantially to 31.1 ± 6.1%, with this difference being statistically significant (mixed model, *P* < 0.001).

In the third deployment, designed to evaluate the influence of floating wetland modules, survival was recorded in enclosures positioned either directly adjacent to or ~50 m away from the floating structures. Survival near the wetlands ranged from 66.7 to 93.3%, with a mean of 81.7%, while survival in the control enclosures far from the wetlands ranged from 80.0 to 96.7%, with a mean of 85.6% (Fig. 3C). An independent *t*-test showed no statistically significant difference between the two treatments (*P* = 0.708).

### Growth

During the first deployment, growth was assessed after a period of 6 weeks. Fish in the East impoundment increased in mean body mass 34.0 ± 1.5%, while fish in the West impoundment experienced a slight decrease of −3.9 ± 4.5% (Fig. 4A). This difference was statistically significant (*t*-test, *P* = 0.001).

**Figure 4:**
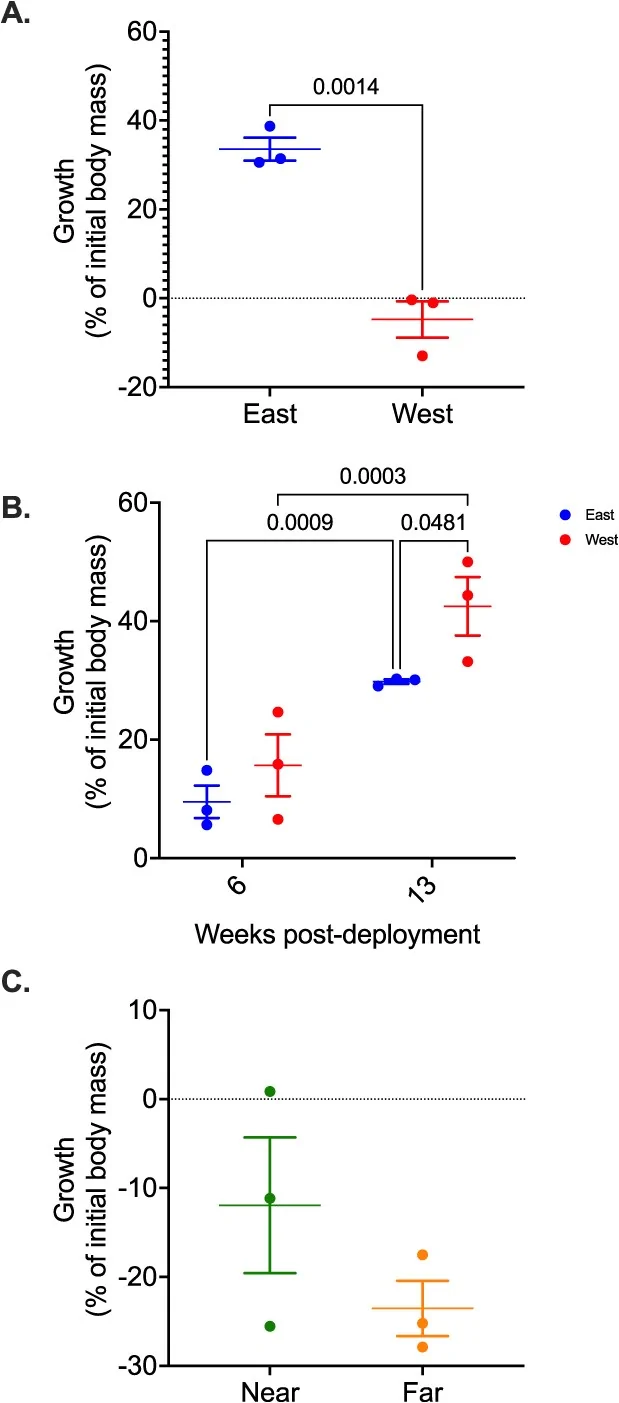
Growth of Delta Smelt across three deployments. Panels A–C depict changes in body mass (percentage of initial mass) observed during each deployment. Panel A shows growth outcomes from the first deployment, comparing fish reared in the East (blue) and West (red) impoundments. Panel B presents growth data from the second deployment, measured at two time points (6 and 13 weeks post-deployment) in the East (blue) and West (red) impoundments. Panel C displays growth data from the third deployment, comparing enclosures located ‘Near’ the floating wetland modules (green) and ‘Far’ from the wetlands (orange) within the West impoundment. Points represent enclosure-level mean growth values, with three replicate enclosures per treatment/time point. Horizontal lines and error bars represent the mean ± SEM calculated across enclosure-level means. Statistical comparisons between groups are annotated with corresponding *P*-values.

In the second deployment, growth was evaluated at two distinct time points: 6 and 13 weeks post-deployment (Fig. 4B). After 6 weeks, fish increased in mass by 10.6 ± 1.9% in the East impoundment and 14.9 ± 3.5% in the West impoundment. By week 13, growth increased to 30.6 ± 2.9% in the East and 44.8 ± 2.9% in the West. Statistically significant differences in growth occurred between the 6- and 13-week time points within both impoundments (two-way ANOVA; East: *P* = 0.001; West: *P* = 0.0003), as well as between the East and West impoundments at week 13 (*P* = 0.048).

For the third deployment, growth was measured at the conclusion of a 4-week period. Fish held near floating wetland modules showed a mean mass reduction of 11.4 ± 5.3%, while fish in control enclosures located far from the wetlands exhibited a mean mass reduction of 20.8 ± 3.4% (Fig. 4C). No statistically significant difference in growth was detected between these two treatments (*t*-test, *P* = 0.249). Fish fork length, wet mass, and calculated condition factor at each sampling point are summarized in Supplementary Table S1.

### Metabolite levels

#### Glycogen

During the second deployment, whole body glycogen concentrations were measured in Delta Smelt at baseline (FCCL hatchery pre-deployment), and subsequently at 6 weeks and at the end of the 13-week deployment period in the East and West impoundments (Fig. 5A). At baseline, glycogen concentrations averaged 14.63 ± 3.38 mg/g (mean ± SEM). Six weeks post-deployment, glycogen levels decreased to 11.71 ± 1.02 mg/g in the East impoundment but slightly increased to 17.48 ± 2.81 mg/g in the West impoundment; this difference between impoundments at 6 weeks was not statistically significant (mixed model, *P* = 0.134). At the final 13-week sampling point, glycogen content significantly declined in the West impoundment (5.79 ± 1.58 mg/g) compared to the East impoundment (10.99 ± 1.41 mg/g; mixed model, *P* = 0.002). Moreover, glycogen concentrations in the West impoundment significantly declined between the 6- and 13-week measurements (*P* < 0.001), whereas the East impoundment showed no significant change over this period (*P* = 0.646).

**Figure 5:**
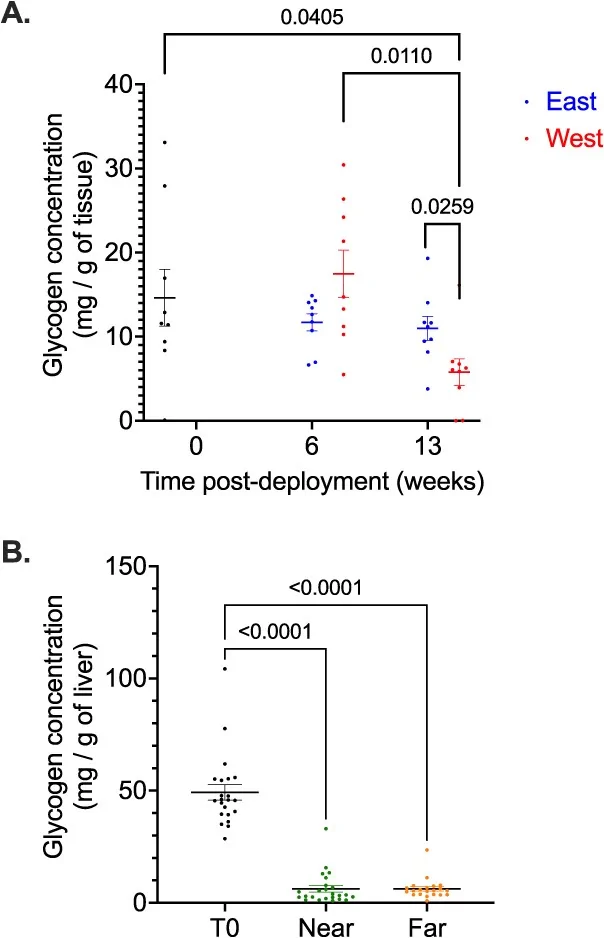
Glycogen content in Delta Smelt during the second and third deployments. Panels A and B show changes in glycogen concentrations (mg/g tissue) measured in individual Delta Smelt at various time points across two field deployments. Panel A presents whole-body glycogen concentrations from the second deployment, measured pre-deployment at the hatchery (T0, black), and at 6 and 13 weeks post-deployment in the East (blue) and West (red) impoundments. Panel B shows liver glycogen concentrations from the third deployment, measured pre-deployment (T0, black) and after a 4-week field exposure in enclosures located ‘Near’ (green) and ‘Far’ (orange) from floating wetland modules within the West impoundment. Points represent individual fish. Horizontal lines and error bars represent the mean ± SEM for each group/time point. Statistical comparisons between groups are annotated with corresponding *P*-values.

For the third deployment, liver glycogen concentrations were assessed at T0 and at the end of the 4-week field deployment (Fig. 5B). T0 fish had a mean liver glycogen concentration of ~49.26 ± 3.47 mg/g. After 4 weeks, fish reared near the floating wetlands had a mean glycogen concentration of 6.21 ± 1.0 mg/g, while those reared far from wetlands had 6.28 ± 1.5 mg/g. Both field-reared groups had significantly lower glycogen levels than the T0 group (Kruskal–Wallis, *P* < 0.0001 for both comparisons), indicating substantial depletion of energy reserves regardless of proximity to the floating wetland habitat. No significant difference was observed between the Near and Far treatments.

#### Cortisol

Whole-body cortisol concentrations were measured in Delta Smelt at the hatchery prior to the second deployment (T0), and again at 6 and 13 weeks post-deployment in the East and West impoundments (Fig. 6). At T0, fish exhibited a mean cortisol concentration of 2.75 ± 0.20 ng/g wet tissue. At 6 weeks post-deployment, cortisol levels were 4.27 ± 1.41 ng/g in the East and 1.74 ± 0.27 ng/g in the West. No significant difference was detected between impoundments at this time point (*P* = 0.182), nor between impoundment and the T0 baseline (East vs T0: *P* = 0.621; West vs T0: *P* = 0.362). At 13 weeks, cortisol concentrations reached 7.95 ± 2.09 ng/g in the East and 16.08 ± 5.17 ng/g in the West. The difference between impoundments was not statistically significant (*P* = 0.174), nor were the comparisons to T0 within impoundments (East: *P* = 0.081; West: *P* = 0.067). However, a mixed-effects model revealed a significant main effect of time on cortisol levels (*P* = 0.0015), indicating that cortisol levels increased significantly over the course of the deployment, irrespective of impoundment. Neither the main effect of impoundment (*P* = 0.3042) nor the interaction between impoundment and time (*P* = 0.055) were significant.

**Figure 6:**
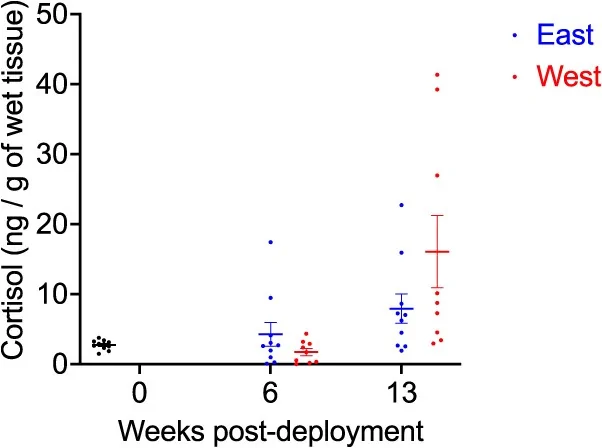
Whole-body cortisol concentrations in Delta Smelt during the second deployment. Data represent whole-body cortisol concentrations (ng/g of wet tissue) measured at three time points: pre-deployment (T0, hatchery baseline, black), and at 6 and 13 weeks post-deployment in the East (blue) and West (red) impoundments. Points represent individual fish. Horizontal lines represent mean cortisol concentrations for each group/time point, and error bars denote the SEM. Statistical analysis revealed a significant main effect of time on cortisol concentrations (mixed-effects model, *P* = 0.002), but no significant differences between impoundments or their interaction with time were observed (*P* > 0.05 for all comparisons).

### Zooplankton

During Deployments 1 and 2 (conducted in the East and West impoundments before installation of floating wetlands), zooplankton communities exhibited reduced temporal and spatial variation in both biomass and taxonomic composition (Fig. 7A). Overall community structure differed between impoundments. In the East impoundment, calanoid copepods accounted for 50.3% of total zooplankton biomass, followed by cyclopoid copepods at 27.4% and cladocerans at 16.7%. In contrast, the West impoundment showed greater dominance by calanoids, which contributed 58.9% of total biomass, with cyclopoids at 25.4% and cladocerans substantially lower at 3.4%. The remaining biomass in both impoundments consisted of ostracods, unresolved copepod taxa or other minor groups. Comparing calanoid communities between impoundments revealed differences in species composition (Fig. 7B). In the East impoundment, the calanoid biomass was more evenly distributed among several taxa: *Arctodiaptomus dorsalis* was most abundant, contributing 21.0% of the total zooplankton biomass, closely followed by unidentified calanoid copepods at 20.2%. *Leptodiaptomus siciloides* was present but comparatively less abundant, accounting for only 5.3% of total biomass. Conversely, the West impoundment exhibited strong dominance by *L. siciloides*, representing 49.7% of total zooplankton biomass and constituting over 90% of the calanoid biomass. This highlights a distinctly more homogeneous calanoid composition in the West impoundment relative to the East. Cyclopoid copepods were consistently dominated by *Acanthocyclops robustus* in both impoundments, contributing 23.4% to total zooplankton biomass, while the remaining biomass within this order (<3%) comprised unidentified or unresolved taxa, likely fragments or immature individuals. The cladoceran community was taxonomically diverse but contributed less overall biomass. *Bosmina longirostris* was the predominant species, accounting for ~7.2% of total biomass across impoundments. Other cladocerans, such as *Daphnia* spp. and minor taxa, contributed an additional ca. 2.9% of total biomass.

**Figure 7:**
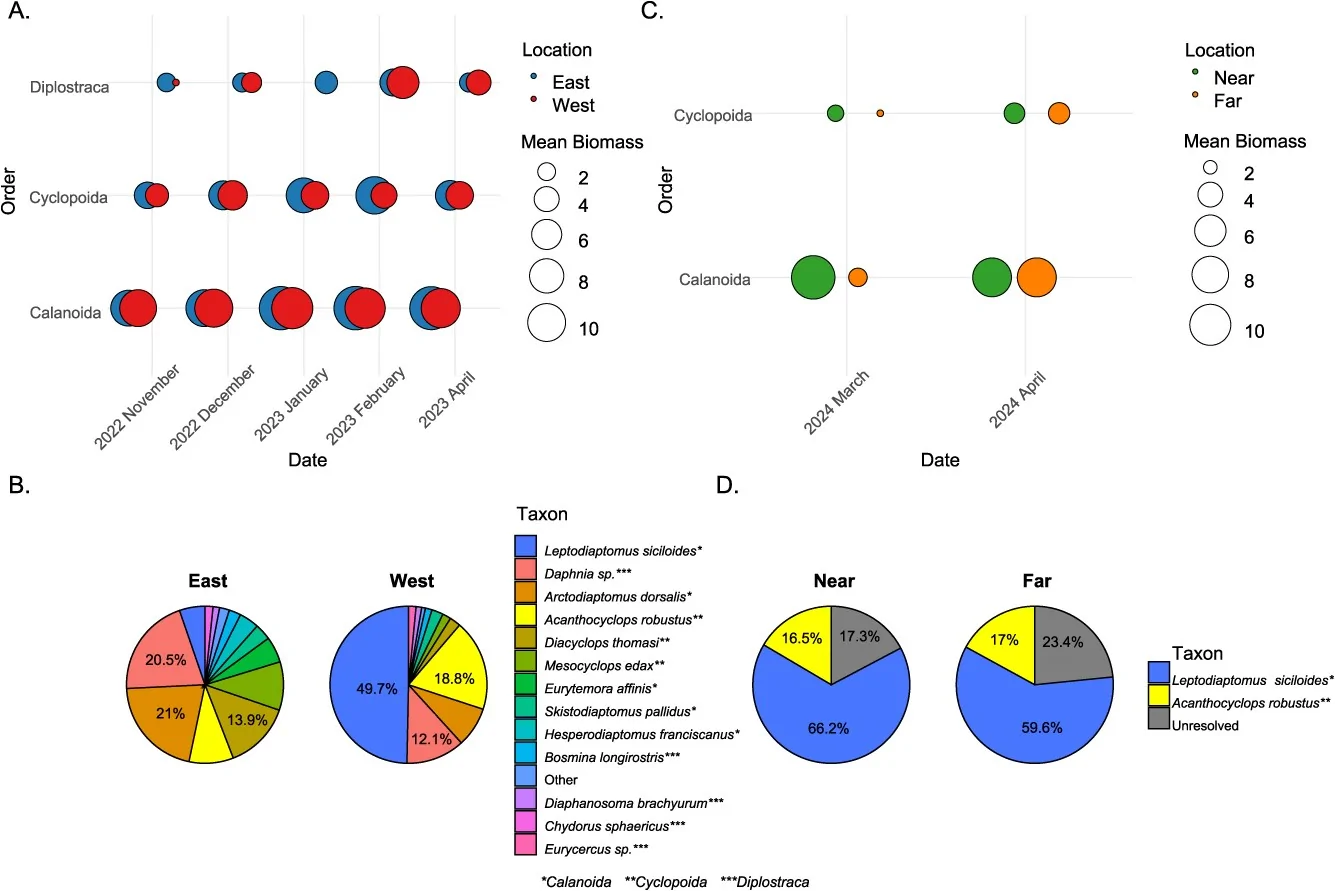
Zooplankton community composition across impoundments and deployments. (A) Relative biomass (%) of major zooplankton groups (calanoid copepods, cyclopoid copepods, cladocerans and others) during Deployments 1 and 2 in the East and West impoundments. (B) Composition of calanoid copepod taxa by relative biomass in the East and West impoundments during Deployments 1 and 2. (C) Relative biomass of major zooplankton groups at Near and Far sites during Deployment 3 (March–April 2024), showing a marked absence of cladocerans and ostracods. (D) Dominant calanoid and cyclopoid copepod taxa by relative biomass during Deployment 3 at Near and Far sites.

During Deployment 3 (March–April 2024) in the West impoundment with floating wetland modules present, zooplankton biomass consisted almost entirely of calanoid and cyclopoid copepods, with no cladocerans or ostracods detected (Fig. 7C). Biomass distributions were similar near and far from the floating wetlands. At the near site, calanoid copepods accounted for 80.0% of total biomass, dominated by *L. siciloides* (66.2%). Cyclopoid copepods, primarily represented by *A. robustus* (16.5%), contributed the remaining 20.0% (Fig. 7D). Unresolved taxa comprised 17.3% of total biomass. Similarly, at the far site, calanoids represented 77.8% and cyclopoids 22.2% of total biomass, with *L. siciloides* (59.6%) and *A. robustus* (17.0%) dominating their respective orders. Unresolved copepod taxa accounted for the remaining 23.4% at this site. These results highlight the pronounced dominance of *L. siciloides* and the limited diversity during this deployment period.

## Discussion

The objective of this study was to assess the feasibility of rearing Delta Smelt (*H. transpacificus*) in managed wetland impoundments and to evaluate whether habitat enhancements, particularly floating wetland modules, could improve survival, growth and physiological performance of captive-reared Delta Smelt in semi-natural environments. Over the course of three deployments on Bouldin Island in the Sacramento–San Joaquin Delta, we monitored key environmental variables and measured survival, growth, glycogen reserves and cortisol levels to determine the suitability of these impoundments for conservation rearing. Additionally, we assessed the zooplankton community composition and availability of prey resources to better understand the foraging environment and food web dynamics supporting Delta Smelt during each deployment. Because deployments differed in season, environmental conditions and fish reproductive status, we first discuss each deployment individually before integrating the broader implications for conservation rearing of Delta Smelt.

### Deployment 1: winter conditions supported high survival and growth

Temperature and DO are critical for Delta Smelt, a species with narrow physiological tolerances (Komoroske *et al.*, 2014, 2015; Jeffries *et al.*, 2016). These factors influence survival, growth, and metabolic performance and are essential to evaluate managed impoundments as rearing habitats. In the winter deployment, water temperatures (ca. 7–16°C) remained within the species’ optimal thermal range, with modest diel variability (Komoroske *et al.*, 2014). Slightly greater temperature variation in the East impoundment could result from depth or exposure differences, but overall, thermal conditions were well suited for Delta Smelt.

Survival was high in both impoundments, exceeding 96% at each site, suggesting that environmental conditions and handling protocols were within the species’ tolerance. Growth patterns were less consistent and appeared to reflect localized differences in water quality and zooplankton community composition between impoundments. Fish in the East impoundment gained weight, whereas fish in the West impoundment lost some. Together, these results suggest that cool winter conditions were sufficient to support high survival in both impoundments, while differences in growth coincided with local variation in water quality and zooplankton community composition, even when acute environmental stress appeared low.

### Deployment 2: late-spring conditions and reproductive constraints

During spring, temperatures increased significantly, peaking at 22.8°C in the East. Although below lethal limits, prolonged exposure above ca. 20°C is associated with reduced aerobic capacity and cellular stress responses, and the species is rarely encountered in the wild at temperatures above ca. 22°C, suggesting that late spring conditions may approach a stress threshold for Delta Smelt (Komoroske *et al.*, 2014, 2015; Jeffries *et al.*, 2016; Pasparakis *et al.*, 2022). This deployment therefore captured a seasonal transition from initially suitable conditions towards a warmer environment in which physiological and energetic demands likely increased.

Survival remained relatively high after 6 weeks but declined sharply by week 13 in the West impoundment, dropping to ~31% compared to 77% in the East. Spawning was observed in the West, with fertilized embryos on mesh enclosures, and post-spawning mortality, consistent with the predominantly annual life cycle and reproductive biology of Delta Smelt, likely contributed to the decline in survival (Bennett, 2005; Hobbs *et al.*, 2019; Kurobe *et al.*, 2022). These observations highlight the importance of considering reproductive timing when planning longer deployments and interpreting survival outcomes. Fish in both impoundments grew over the course of the deployment, with larger gains in the West, where reduced densities after spawning mortality may have lowered competition for food. This apparent growth advantage among surviving fish should therefore be interpreted within the context of concurrent mortality, reproductive activity and changing fish density rather than as evidence that late-spring conditions were broadly favourable.

Whole-body glycogen responses during Deployment 2 suggest that short-term carbohydrate reserves were increasingly drawn down as spring conditions progressed, with the strongest depletion observed in the West impoundment by week 13. Glycogen reserves were relatively high in hatchery fish at T0 and remained broadly similar after 6 weeks in the field, indicating that early-spring exposure did not immediately deplete this reserve. By week 13, however, fish in the West showed marked glycogen depletion, whereas levels in the East remained comparatively stable. This decline coincided with observed spawning activity and is consistent with increased energetic demand associated with reproduction (Bennett, 2005; Kurobe *et al.*, 2022). Rising water temperatures may have further increased metabolic demand and glycogen use, particularly when paired with reproductive effort and possible food limitations. Interpreted alongside survival, growth and cortisol responses, these glycogen patterns provide one physiological signal of increasing challenge during the longer spring deployment.

Whole-body cortisol concentrations increased over the course of the deployment, indicating a cumulative rise in physiological challenge over time. Cortisol levels were low at the hatchery baseline and remained relatively low after 6 weeks in both impoundments, suggesting that early spring conditions were not acutely stressful, likely due to moderate temperatures, stable oxygen levels and adequate prey availability. By week 13, however, cortisol concentrations had increased in both impoundments. Because pairwise differences between sites were not statistically significant, this response should be interpreted primarily as a time-related increase in physiological stress rather than a clear impoundment-specific effect. This increase coincided with seasonal warming, glycogen depletion, changing fish density, and, in the West, the added energetic cost of spawning and potential senescence of post-spawning adults. Elevated temperature is a well-established stressor for Delta Smelt, affecting performance, energetic status and cellular pathways related to heat shock proteins, immune function and homeostasis (Komoroske *et al.*, 2015; Jeffries *et al.*, 2016; Pasparakis *et al.*, 2022). Pasparakis *et al.* (2023) further demonstrated that combined exposure to thermal stress and low turbidity conditions results in elevated cortisol and altered behaviour, underscoring the species’ sensitivity to compounded environmental challenges. Although cortisol concentrations showed a stronger upward trend in the West impoundment by week 13, these differences were not statistically significant and may reflect the combined effects of warming, reproductive effort, declining energy reserves and changing fish density rather than a single site-specific stressor.

### Deployment 3: early-spring evaluation of floating wetland modules

During the third deployment, temperatures increased from ca. 13°C to nearly 20°C over 4 weeks. Diurnal temperature swings were smaller near the floating wetlands (1.99 ± 0.17°C) than far from them (2.50 ± 0.19°C), likely due to shading and wind reduction. This thermal buffering could be relevant for Delta Smelt because rapid temperature changes can contribute to physiological stress (Komoroske *et al.*, 2014; Jeffries *et al.*, 2016). While temperature remained within sublethal bounds, DO levels showed more variability. The near-wetland site experienced 8.5 cumulative hours below 3 mg/l across 11 events; the far site experienced nearly triple that duration. Floating wetlands appeared to dampen DO fluctuations, potentially through shading effects that altered local biological activity. This deployment therefore tested whether habitat structure could buffer environmental variability during a warming early-spring period.

Survival remained high, with no significant difference between fish reared near floating wetlands and those far from them, despite multiple episodes of low DO. Fish mass declined in both treatments, with a smaller decline near the floating wetlands; although not significant, this pattern is consistent with modest buffering by the added structure. Together, these results suggest that floating wetlands may have moderated local environmental variability, but that the magnitude of this buffering was not sufficient to produce clear improvements in survival or growth during this deployment.

Liver glycogen, which reflects short-term energy reserves, declined sharply over the 4-week deployment in all fish from ca. 49.3 to ca. 6.2 mg/g, with no difference between treatments. This decline could indicate energetic stress, particularly if prey availability, prey quality or water-quality conditions limited energy intake. However, similar values have been reported in wild-caught Delta Smelt (Dhayalan *et al.*, 2024), suggesting that low liver glycogen may also reflect elevated energy turnover associated with natural foraging conditions rather than poor condition alone. Hatchery baselines, elevated by predictable feeding and low activity, are therefore unlikely to represent energetic patterns expected under natural conditions. At the same time, low glycogen in wild fish may reflect widespread energetic stress in a depleted population; our comparison does not, by itself, distinguish acclimatization from stress. This interpretation is consistent with broader ecological theory, as maintaining large energy stores can be costly when fish must increase foraging effort and potentially elevate predation risk (Lima and Dill, 1990; Witter and Cuthill, 1993). Because fish in this third deployment were not migrating or spawning, the observed glycogen depletion is consistent with a shift towards more realistic energy use under semi-natural conditions, although periods of food limitation or suboptimal water quality may also have contributed.

Overall, the third deployment suggests that floating wetland modules may provide modest environmental buffering, but that these physical changes did not translate into detectable improvements in survival, growth or liver glycogen over the duration of this study.

### Cross-deployment synthesis and management implications

Across all three deployments, Delta Smelt performance within managed impoundments appeared to depend on the balance among environmental stability, energetic demand and prey quality. Cooler winter and early-spring conditions generally supported high survival, whereas prolonged warming later in the season, particularly when coupled with episodic low DO events, coincided with declining rearing conditions and greater energetic challenge. These patterns suggest that deployment timing will be a central consideration for using managed impoundments as semi-natural rearing habitat but also highlight the potential value of habitat-management approaches that moderate temperature and DO variability during warmer periods, consistent with field-deployment studies showing that Delta Smelt survival, growth and physiological condition vary across seasons and environmental contexts (Davis *et al.*, 2024).

The physiological responses measured here suggest that semi-natural rearing produced both potential signs of acclimatization and evidence of constraint. Glycogen depletion may partially reflect a transition towards more natural energetic demands under semi-natural foraging conditions. In this context, moderate glycogen depletion may indicate adjustment to more realistic energy use rather than poor condition alone, particularly because similarly low glycogen values have been reported in wild-caught Delta Smelt (Dhayalan *et al.*, 2024). However, stronger glycogen depletion, particularly when paired with increasing cortisol concentrations, suggests that fish may experience physiological stress as conditions become less favourable. Because cortisol reflects cumulative physiological challenge rather than any single stressor, the elevated concentrations observed during the late-spring deployment likely resulted from the combined effects of warming temperatures, reproductive effort, prey limitations, changing fish density and declining energy reserves. Additional measures of energetic condition, including lipid and protein reserves, would help distinguish acclimatization from energetic stress in future studies. This distinction is important for conservation rearing because semi-natural exposure may help hatchery-reared fish adjust to wild prey fields and variable environmental conditions, but maintaining sufficient energetic reserves before release may also provide an important buffer during the transition to the wild.

Zooplankton community composition across the three deployments revealed differences in prey availability with important implications for Delta Smelt foraging success, growth and energetic condition. During the first two deployments, calanoid copepods dominated zooplankton biomass in both impoundments, although species composition varied. The East impoundment exhibited a more taxonomically diverse calanoid assemblage, including *A. dorsalis*, while the West impoundment was strongly dominated by *L. siciloides*, which alone accounted for nearly half of total zooplankton biomass. This dominance persisted into the third deployment, where *L. siciloides* made up over 60% of biomass at both Near and Far sites. Delta Smelt are visual planktivores that preferentially target large-bodied, slow-moving calanoid copepods such as *Pseudodiaptomus forbesi* (ca. 1.2–1.5 mm), *Eurytemora affinis* (ca. 1.0–1.6 mm), and *Sinocalanus doerrii* (ca. 0.9–1.4 mm), which provide higher caloric content and are easier to capture (Moyle *et al.*, 1992; Hobbs *et al.*, 2006; Slater and Baxter, 2014). In contrast, *L. siciloides* is smaller (ca. 0.4–0.7 mm) and more evasive, making it a suboptimal prey species for Delta Smelt. Cyclopoid copepods, especially *A. robustus*, were also abundant in both impoundments, comprising up to 25% of biomass. Although *Acanthocyclops* spp. have occasionally been detected in Delta Smelt gut contents (Hobbs *et al.*, 2006), it is not considered a preferred prey item, likely due to its small size, rapid escape responses and lower energetic value. The absence of cladocerans and other alternative prey taxa during the third deployment further reduced the diversity of foraging opportunities. Although zooplankton biomass was relatively high, limited prey quality and diversity would be expected to reduce net energy intake. In parallel, spawning during the late-spring deployment likely imposed substantial additional energetic demands; together, limited prey quality and reproductive effort provide a plausible mechanistic explanation for the variable growth responses, glycogen depletion and increasing physiological challenge observed across deployments. Because zooplankton sampling was conducted at the impoundment scale rather than directly within enclosures, future studies could evaluate enclosure-specific prey availability to better link prey dynamics with fish performance. Coupling enclosure-specific prey sampling with gut content and trophic analyses would further help determine whether available prey communities translate into effective prey utilization and energy assimilation by Delta Smelt under semi-natural rearing conditions. Given the small size and hydraulic controllability of managed impoundments, targeted prey enhancement strategies, including supplementation with preferred calanoid copepods or managed fertilization approaches designed to increase zooplankton production, may represent promising tools for improving rearing performance under semi-natural conditions.

## Conclusion

This study demonstrates that managed wetland impoundments can function as semi-natural rearing environments for Delta Smelt prior to supplementation, while habitat enhancements such as floating wetlands may provide modest environmental buffering. Across three seasonal deployments, Delta Smelt demonstrated high survival under most conditions, successfully foraged on wild prey, and exhibited physiological responses, such as reductions in glycogen reserves and elevations in cortisol that, while reflecting increased environmental challenge, may also be consistent with acclimatization to natural variability. However, the observed declines in energy reserves and increased stress physiology during spring months highlight the importance of timing, water quality management and prey availability in improving impoundment performance. These results suggest that managed impoundments may be most suitable for Delta Smelt rearing during cooler winter and early spring conditions, when temperature and DO remain within favourable ranges. While the dominant zooplankton taxa present in the impoundments were not optimal for Delta Smelt, the controlled nature of these systems presents a unique opportunity to actively manage food webs. Future work could test whether seeding impoundments with preferred prey species such as *P. forbesi*, *E. affinis* and *S. doerrii* or using managed fertilization approaches to enhance zooplankton production can improve foraging success and physiological condition.

Together, these findings highlight the value of impoundments as transitional habitats that bridge the gap between captivity and the wild, exposing fish to more natural environmental and foraging conditions while maintaining a degree of protection and management control. Future studies could also compare impoundment-reared and hatchery-reared fish in release-relevant assays, including hypoxia tolerance, thermal susceptibility, foraging performance and anti-predator behaviour. In addition, larger enclosures containing predator-cue or predator-accessible zones and refuge areas could be used as pre-release training environments to test whether controlled predator exposure improves predator recognition and avoidance behaviour in Delta Smelt while maintaining safe areas for acclimatization. Continued refinement of habitat features, food web management and seasonal deployment strategies will be essential for realizing the full potential of conservation aquaculture in Delta Smelt recovery efforts.

## Supplementary Material

Web_Material_coag076

## Data Availability

Data supporting the findings of this study are available from the corresponding author upon reasonable request.
