# Supplementary material for: Beyond the hatchery: testing managed wetland impoundments as semi-natural rearing habitat for endangered Delta Smelt
Source: Conserv Physiol. 2026 Oct 1;14(1):coag076. doi: 10.1093/conphys/coag076 (PMC13630425; doi:10.1093/conphys/coag076)
Supplement: Supplementary file 1 [file web_material_coag076.zip › Supplemental Table S1.pdf]

Deployment	Sampling point	Group / treatment	Fork length (mm)	Wet mass (g)	Condition factor K
Deployment 1	T0 / hatchery	Hatchery baseline	47.8 ± 0.7	1.03 ± 0.22	0.800 ± 0.06
Deployment 1	End of deployment	West impoundment	53.9 ± 0.7	0.98 ± 0.04	0.606 ± 0.010
Deployment 1	End of deployment	East impoundment	57.4 ± 1.1	1.37 ± 0.09	0.697 ± 0.017
Deployment 2	T0 / hatchery	Hatchery baseline	60.1 ± 0.7	1.93 ± 0.15	0.909 ± 0.077
Deployment 2	6 weeks	East impoundment	65.4 ± 0.8	2.09 ± 0.09	0.732 ± 0.017
Deployment 2	6 weeks	West impoundment	65.1 ± 1.0	2.24 ± 0.09	0.804 ± 0.018
Deployment 2	13 weeks	East impoundment	68.7 ± 0.6	2.51 ± 0.06	0.771 ± 0.012
Deployment 2	13 weeks	West impoundment	70.8 ± 1.2	2.77 ± 0.15	0.771 ± 0.016
Deployment 3	T0 / hatchery	Hatchery baseline	73.2 ± 1.5	3.27 ± 0.22	0.801 ± 0.017
Deployment 3	End of deployment	Near	73.5 ± 1.3	2.82 ± 0.18	0.695 ± 0.020
Deployment 3	End of deployment	Far	69.4 ± 1.3	2.39 ± 0.20	0.684 ± 0.025
